# Predicting patient use of general practice services in Australia: models developed using national cross-sectional survey data

**DOI:** 10.1186/s12875-019-0914-y

**Published:** 2019-02-14

**Authors:** Christopher Harrison, Joan Henderson, Graeme Miller, Helena Britt

**Affiliations:** 1 Menzies Centre for Health Policy, School of Public Health, University of Sydney, Level 6, Charles Perkins Centre, Camperdown, Australia; 2 (Then) Family Medicine Research Centre, School of Public Health, University of Sydney, Camperdown, Australia

## Abstract

**Background:**

The ageing population and increasing prevalence of multimorbidity place greater resource demands on the health systems internationally. Accurate prediction of general practice (GP) services is important for health workforce planning. The aim of this research was to develop a parsimonious model that predicts patient visit rates to general practice.

**Methods:**

Between 2012 and 2016, 1449 randomly selected Australian GPs recorded GP-patient encounter details for 43,501 patients in sub-studies of the Bettering the Evaluation and Care of Health (BEACH) program. Details included patient characteristics, all diagnosed chronic conditions per patient and the number of GP visits for each patient in previous 12 months. BEACH has a single stage cluster design. Survey procedures in SAS version 9.3 (SAS Inc., Cary, NC, USA) were used to account for the effect of this clustering. Models predicting patient GP visit rates were tested. R-square value was used to measure how well each model predicts GP attendance. An adjusted R-square was calculated for all models with more than one explanatory variable. Statistically insignificant variables were removed through backwards elimination. Due to the large sample size, *p* < 0.01 rather than *p* < 0.05 was used as level of significance.

**Results:**

Number of diagnosed chronic conditions alone accounted for 25.48% of variance (R-square) in number of visits in previous year. The final parsimonious model accounted for 27.58% of variance and estimated that each year: female patients had 0.52 more visits; Commonwealth Concessional Health Care Card holders had 1.06 more visits; for each chronic condition patients made 1.06 more visits; and visit rate initially decreased with age before increasing exponentially.

**Conclusions:**

Number of diagnosed chronic conditions was the best individual predictor of the number of GP visits. Adding patient age, sex and concession card status explained significantly more variance. This model will assist health care planning by providing an accurate prediction of patient use of GP services.

## Background

The ageing of the population and an increasing prevalence of multimorbidity, are expected to place greater demands on the Australian health system [1–3]. Being able to accurately predict patient use of general practice services is important for health workforce planning.

Traditionally, in Australia, a simple ratio of full time equivalent general practitioners (GPs) to population has been used to estimate adequacy of GP supply for a geographic area [4]. However this method fails to consider differing levels of health care demand by different types of patients. A patient’s age and sex have been shown to influence the length of their GP encounters [5] and the number of times they see a GP in a year [2]. For example, on average, an 85 year old male patient will spend 291 min with a GP over a year while his 12 year old granddaughter will spend only 28 min [2]. This variance is important as inner regional areas of Australia have higher proportions of older residents than other areas and therefore have higher demand for GP services than an average GP:population ratio would estimate [2]. The ability to predict patient demand would improve the accuracy of policy planners’ projections of required GP workforce.

Australian GPs are paid on a fee-for-service basis, covered (fully or in part) by a universal health insurance scheme called Medicare. GP remuneration is primarily based on the number of times they see patients. The Australian Federal Government is trialling ‘Health Care Homes’ in which GPs will receive capitation payments for managing the chronic conditions (but not non-chronic conditions) of enrolled patients [6]. The trial commenced on 1st October 2017 and will conclude on 30th November 2019. Ideally, the capitation payment should at least reflect the amount the GP would have earned through fee-for-service for managing that patient. Each patient will be assigned to one of three tiers of “complexity and need” with higher GP remuneration for care of those in higher tiers ($591 tier 1, $1267 tier 2 and $1795 tier 3) [6]. However, there is concern that the planned tier assignment tools may not accurately reflect patient demand for GP care [7]. If it does not, GPs may choose not to enrol in the program, or those who do may only enrol patients with relatively low demand for services. The trial has experienced lower than expected uptake by patients and a substantial number of practices have withdrawn from the trial [8, 9]. An accurate measure of patient demand would provide a structure on which an appropriate reimbursement for GPs could be calculated.

In 2000, Knox et al. found a range of patient characteristics were associated with the number of times a patient sampled at a GP encounter had seen a GP in the previous year [10]. After adjustment for other factors, characteristics related to visit rate were: patient age (older patients visiting more often); holding a Commonwealth Concession Health Care Card (CCHCC) (attended more often) and number of chronic conditions (increase in visits for every additional chronic condition). Patient sex was not independently associated.

Since Knox’s study, the population has aged considerably, with a corresponding increase in the number of GP consultations with patients aged 65 years or older [11]. To better identify future demand, geographic areas of need and appropriate capitation payments, a scientifically based tool is required to predict patient demand for GP services. We therefore examined known predictors of patient use of GP services with a particular focus on the number of diagnosed chronic conditions in an individual patient. For ease of reading, we will refer to ‘diagnosed chronic conditions’ simply as ‘chronic conditions’.

Multimorbidity is the term commonly used to describe patients with multiple chronic conditions [12]. While multimorbidity has commonly been measured by counting the number of individual conditions, some researchers believe there are advantages in counting the number of ‘groups’ of similar conditions [12]. Examples of ‘groups’ of conditions are the domains of the Cumulative Illness Rating Scale (CIRS) [13], the chapters of the International Classification of Primary Care Version 2 (ICPC-2) [14] or those of the International Classification of Diseases Version 10 (ICD 10) [15]. Previous research has shown that using CIRS domains, ICPC-2 or ICD-10 chapters, the same patients were identified as having three or more domains/chapters with at least one chronic condition in each [12]. Using groups of conditions may improve reliability of results. For instance, two inter-related conditions (e.g. chronic ischaemic heart disease and myocardial infarction) may be recorded as two separate conditions by one clinician while another may consider them to be a single entity. Only counting the body system of these conditions once would ameliorate labelling inconsistency. In another scenario, a condition such as hypertension, that over time develops into complicated hypertension, might then receive a slightly different label or code in the medical record. Once again, counting only the body system to which the conditions were classified would remove the double-count.

It has been argued that the diagnosis of a chronic condition in a body system previously free of any condition will have a greater impact on the patient’s care than the diagnosis of an additional chronic condition in a body system. [12] This is because chronic conditions in different body systems are more likely to compete for treatment, while treatments for those in the same system are more likely to be complementary [12]. While the number of chronic conditions has been shown to be a predictor of patient visits to GPs, [8] the predictive value of the number of different body systems has yet to be tested. In future we will refer to the concept of ‘body systems with at least one chronic condition classified’ simply as ‘body systems’.

In a separate analysis Knox et al. examined the effect of each of nine prevalent individual chronic conditions [10]. After accounting for other significant variables, including total number of chronic conditions, they found that patients attended more often if they had depression, anxiety or chronic back pain. Ideally, a wider range of chronic conditions should be tested for their independent effect on visit rate.

Some researchers believe that both the number of chronic conditions and the severity of illness are important [16, 17]. The CIRS [13] is a widely used example that includes severity of illness in the measurement of multimorbidity. Without accounting for severity of illness, a patient with well controlled hypertension, hyperlipidaemia and mild asthma (and no others), and another patient with the same conditions, but severe and uncontrolled, would be considered to have the same level of multimorbidity. However, whether a patient severity of illness is an independent predictor of GP use has not yet been established.

The aim of this study is to develop a parsimonious model that predicts patient visit rate to GPs, by examining the predictive power of:

- the number of chronic conditions.
- the number of body systems. This is to test whether a count of body systems is as good a predictor as a count of individual conditions.
- patient age and sex
- the patient characteristics examined by Knox et al. [10] with the addition of number of body systems
- the presence of specific chronic conditions. We will examine a wider range than Knox et al. [10]
- overall severity of patient illness.

## Methods

These data were collected through a series of sub-studies of the BEACH program [18]. BEACH was a continuous, national cross-sectional study of Australian general practice activity running from April 1998 to March 2016. Full methods of the BEACH program are described in detail elsewhere [18]. In summary, each year an ever-changing, random sample of about 1000 GPs participated, each recording information about the content of encounters with 100 consecutive consenting patients, on structured paper forms. Verbal consent was obtained from the GPs at time of recruitment by the research team. Each participating GP obtained verbal consent from participating patients at the start of the recorded encounter, and noted in the patient’s record that they were a BEACH participant for that encounter only. Patients were never identified to the research team. However, for both GPs and patients, variables such as date of birth and postcode are replaced with age categories and general geographic location whenever data are released publicly to prevent the potential for identification where communities are small. These procedures were requested and approved by the Human Research Ethics Committee of the University of Sydney.

BEACH sub-studies allowed for collection of patient-based data not necessarily related to the encounter. The methods for this sub-study are described in greater detail elsewhere [9]. In brief, 1800 participating GPs over twelve five-week recording periods between 27th November 2012 and 28th March 2016, collected information on each of a preordained 30 consecutive patients within their 100 BEACH encounter forms. GPs recorded the number of times the patient had seen any GP in the previous 12 months (including the recorded visit) and all diagnosed chronic conditions in that patient. For ease of completion, tick boxes were provided for 28 prevalent chronic conditions and additional blank spaces were supplied for free text recording of other chronic conditions. The order of listed chronic conditions was changed throughout the sub-studies to reduce any order effect bias. Examples of the instruction sheet and the recording form provided to the GPs have been published elsewhere [18]. The final question varied over the sub-studies. For two of the 18 sub-studies the GPs were also asked to rate the patient’s overall severity of illness (based on their clinical opinion) using a 0–10 point Likert scale where ‘0’ is least and ‘10’ is most, severe.

### Data analysis

Where number of GP visits in previous year was not recorded, the patient was assigned the average number of visits for patients of the same sex, in the same 10-year age group, with the same number of chronic conditions (0,1,2,3+ chronic conditions).

The likelihood of a patient being sampled was directly related to how often they visited a GP. Frequent attenders were more likely to be sampled than infrequent attenders, since they account for more GP encounters. We adjusted for low or high attenders by weighting each patient’s data by the number of times they were said to have seen a GP in the previous year, with low attenders being weighted up and high attenders being weighted down. The resulting weighted data set represents those patients who visited a GP at least once in the previous year, which we will call ‘active patients’.

Patient Indigenous status included patients who self-identified as Aboriginal and/or Torres Strait Islander. A patient’s relative level of advantage/disadvantage was determined using the Australian Bureau of Statistic’s (ABS) Index of Relative Socio-Economic Advantage and Disadvantage (IRSAD) [19], patient residential postcodes in the lower 5 deciles being considered ‘Disadvantaged’ and postcodes in the upper 5 deciles considered ‘Advantaged’. Patient rurality was defined using the ABS‘s Australian Statistical Geography Standard (ASGS) [20], their residential postcode being classified ‘Major city’ or ‘Regional/remote’.

Different body systems were represented using ICPC-2 Chapters [14]. A body system was only counted once per patient, even if the patient had multiple chronic conditions classified to one body system.

Table 1 shows the models we tested and the initial explanatory variables for each model. The number of times patients saw a GP in the previous year was the outcome for all models. The R-square value was used to measure how well each model predicts GP attendance. An adjusted R-square was calculated for all models with more than one explanatory variable. Previous results on the relationship between age and patient GP visit rate showed that the rate decreased from very young patients to adolescents before increasing steadily with older age, [2] suggesting likelihood that the relationship was quadratic in nature. This was also tested (i.e. age^2^).

**Table 1:** Initial variables included in models tested

Model Number	Variables initially included in models as explanatory variables	
Model 1	Number of chronic conditions	
Model 2	Number of body systems (ICPC-2 chapters)	
Model 3	Age, Age^2^ and sex	
Model 4	Number of chronic conditions	
	Number of body systems	
	Age Age^2^ Sex	
	Indigenous status (self-identified)	
	Level of relative disadvantage/advantage (1–5 and 6–10 on IRSAD)	
	Major city Vs regional/remote area (ASGC)	
	Commonwealth Concession Health Care Card (CCHCC) holder	
Model 4A	All variables from Model 4 with the addition of the presence/absence of each the following:	
	Anxiety	Asthma
	Atrial fibrillation	Chronic back pain
	Chronic renal failure	Chronic obstructive pulmonary disease
	Congestive heart failure	Dementia
	Depression	Gastroesophageal reflux disease
	Glaucoma	Hyperlipidaemia
	Hypertension	Hyperthyroidism
	Hypothyroidism	Insomnia
	Ischaemic heart disease	Malignant neoplasm
	Obesity	Osteoarthritis
	Osteoporosis	Other arthritis
	Peripheral vascular disease	Rheumatoid arthritis
	Sleep apnoea	Stroke/cerebrovascular accident
	Type 1 diabetes	Type 2 diabetes
**`**	All variables from Model 4 with the addition of: Patient overall severity of illness	

Statistically insignificant variables were removed through backwards elimination. Due to the large sample size, we used *p* < 0.01 rather than *p* < 0.05 as our level of significance. Any variable removed that had a significance of p < 0.05 will be reported in the text.

Models 4A and 4B were extensions of Model 4. In Model 4A, the presence/absence of each of the 28 common chronic conditions listed on the recording form was added. In Model 4B we added GP assessment of patient severity of illness from the sub-sample previously described. As the data for Model 4B is a subset of the data used in the original test of Model 4, we first retested Model 4 with only this subset to ensure that any changes in variables retained in Model 4B (compared with Model 4) were the result of inclusion of severity of overall illness.

BEACH sub-studies have a single stage cluster design, with each GP having 30 patients clustered around them. Survey procedures in SAS version 9.3 (SAS Inc., Cary, NC, USA) were used to account for the effect of this clustering.

## Results

Completed recording forms were returned by 1449 GPs of the 1800 (80.5%) recruited. There were 43,501 patients in this sample, of whom 41,722 (95.9%) had a reported number of GP visits in the previous year. These patients had an average 9.66 GP visits in that time, and after weighting, we estimated that active patients had an average 4.54 GP visits over the previous 12 months.

The age-sex distribution of the sample is reported elsewhere [7]. In summary it was similar to that of patients at all Medicare or Department of Veteran Affairs (DVA) claimed GP consultations (precision ratio range 0.80–1.14). After weighting for each patient’s attendance over the previous year to create our ‘active patients’ sample, the age-sex distribution was similar to that of all patients who had claimed at least one Medicare GP service item in the previous year.

### Model 1

**Fig. 1:**
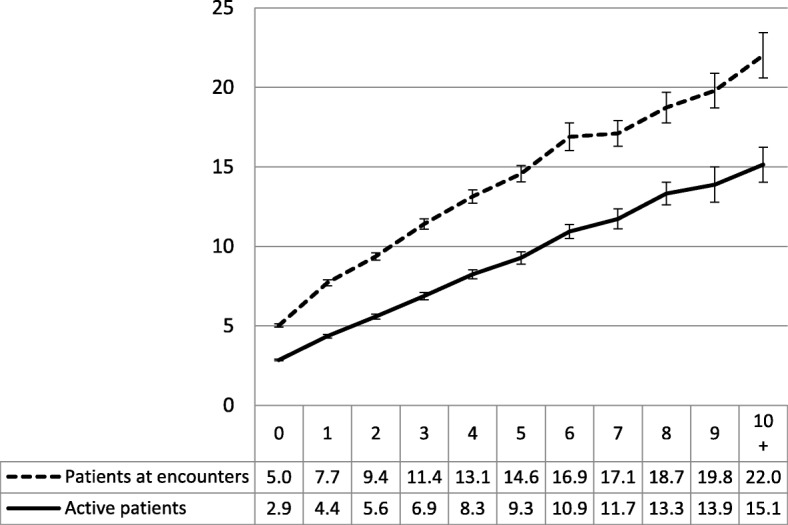
Number of GP visits in previous year by number of individual chronic conditions (95% CIs). Y axis label = Number of GP visits in a year. X axis label = Number of individual chronic conditions. Footnotes = Linear regression model. Number of chronic conditions = Number of GP visits in a year. Patients at encounter R-square = 20.36% (*p*<0.0001). Active patients R-square = 25.48% (*p*<0.0001)

Among sampled patients the number of GP visits in the previous 12 months (visit rate) significantly increased with the number of chronic conditions, from 5.0 visits for sampled patients with no chronic conditions to 22 visits for those with 10 or more. For active patients, the average visit rate increased from 2.9 for those with no chronic conditions to 15.1 for those with 10 or more. A simple linear regression model found that the number of chronic conditions alone accounted for 20.36% of all the variance (R-square) in the visit rate of patients at encounters and 25.48% of the variance among active patients (Fig. 1).

### Model 2

**Fig. 2:**
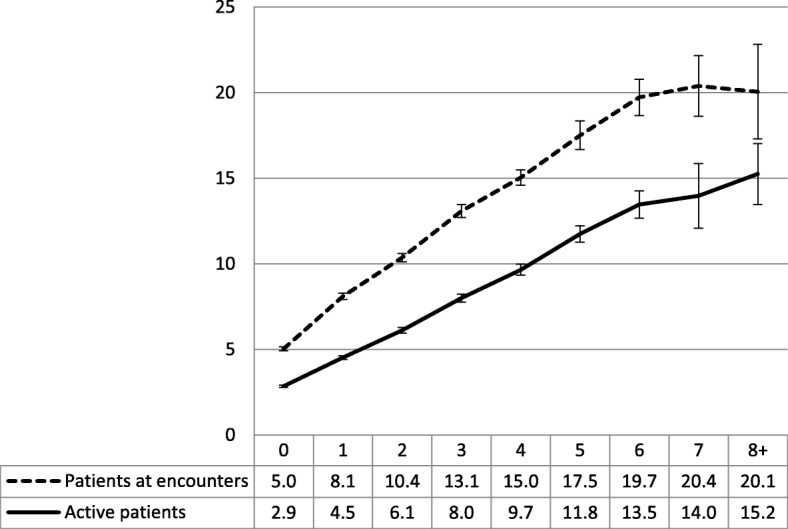
Number of GP visits in previous year by number of body systems (95% CIs). Y axis label = Number of GP visits in a year. X axis label = Number of body systems. Footnotes = Linear regression model. Number of body systems = Number of GP visits in a year. Patients at encounter R-square = 18.77% (*p*<0.0001). Active patients R-square = 23.91% (*p*<0.0001)

The GP visit rate increased with the number of body systems, from 5.0 visits for sampled patients with no chronic conditions to 20.1 visits for those with at least one condition in eight or more different body systems. For active patients, the average visit rate increased from 2.9 for those with no chronic conditions to 15.2 for those with chronic conditions in eight or more different body systems. A simple linear regression model found that the number of body systems accounted for 18.77% of all the variance (R-square) in the GP visit rate of sampled patients and 23.91% of the variance among active patients (Fig. 2).

### Model 3

**Fig. 3:**
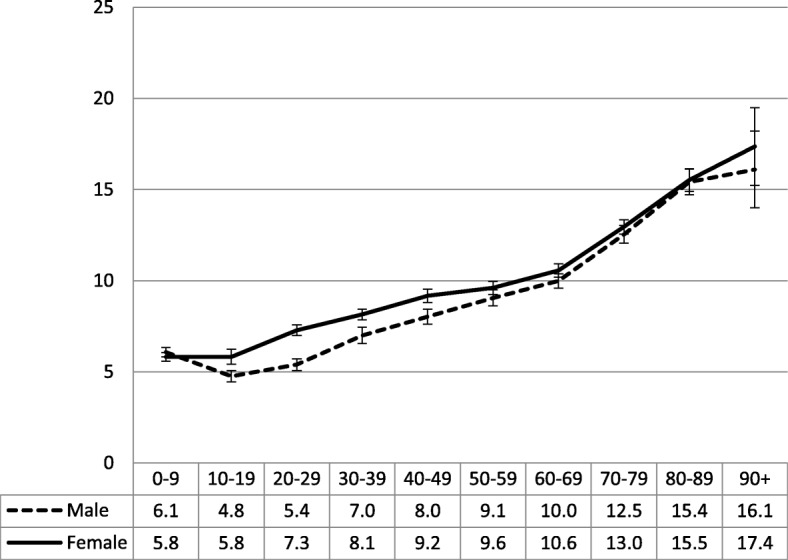
Number of GP visits in previous year by sampled patient age and sex (95% CIs). Y axis label = Number of GP visits in a year. X axis label = Age group (years). Footnotes = Patient age and sex = Number of GP visits in a year. Patients at encounters: R-square = 9.24% (*p*<0.0001); Adjusted R-square = 9.23%. Patient sex, age, and age^2^ = Number of GP visits in a year. Patients at encounters: R-square = 10.15% (*p*<0.0001); Adjusted R-square = 10.14%

For each of the four-decade age-groups from 10 to 49 years, sampled female patients had a significantly higher GP visit rate than male patients. Among females, the visit rate increased significantly with age, especially after the 60–69 years age group. Among male patients the visit rate decreased between the 0–9 and 10–19 years age groups, but then increased significantly with age. A regression model found that the sex of patient and the age of patient accounted for 9.24% of the variance in the visit rate of sampled patients. When age^2^ was added to the model, the amount of variance explained increased to 10.15% (Fig. 3).

**Fig. 4:**
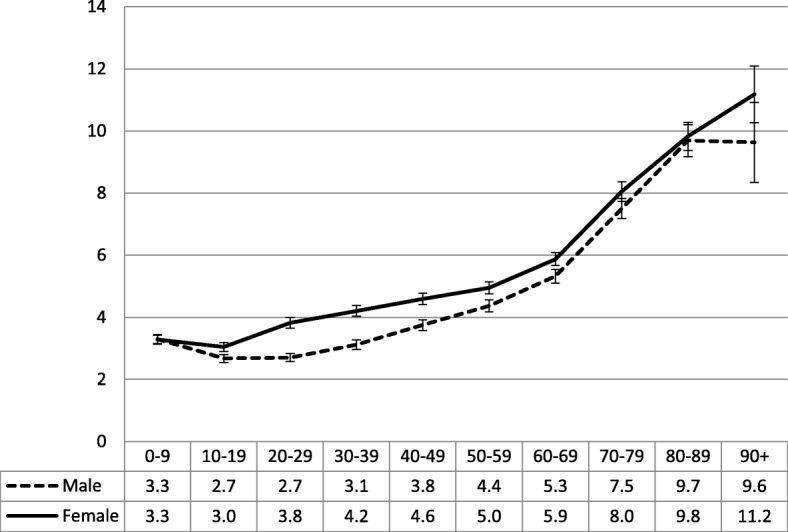
Number of GP visits in previous year - active patients by age and sex (95% CIs). Y axis label = Number of GP visits in a year. X axis label = Age group (years). Footnotes = Patient age and sex = Number of GP visits in a year. Active patients: R-square = 11.23% (*p*<0.0001); Adjusted R-square = 11.22%. Patient sex, age, and age^2^ = Number of GP visits in a year. Active patients: R-square = 14.28% (*p*<0.0001); Adjusted R-square = 14.27%

The pattern for active patients was similar. For the six-decade age groups from 10 to 69 years, female active patients had significantly higher visit rates on average than active male patients (Fig. 4). From the age group of 10–19 years, the GP visit rate increased significantly with age for female active patients. For active male patients the visit rate decreased between the age groups of 0–9 years and 10–19 years before increasing with older age. A regression model showed that the sex and the age of patients accounted for 11.23% of all the variance in GP visit rate for active patients. When age^2^ was included in the model, the variance explained increased to 14.28%. The adjusted R-square of this model was very similar at 14.27% (Fig. 4).

### Model 4

Through backward elimination, patient Indigenous status, patient relative advantage/ disadvantage, number of body systems, and patient rurality were removed. Patient rurality was the last to be removed, with a *p*-value of 0.0284 and an effect size of 0.334 more visits for major city patients. The final model accounted for 27.59% of the variance in GP visit rate. The adjusted R-square was very similar (27.58%). After adjusting for all other significant variables: female patients had about half a visit (0.52) more per year than male patients; those with a CCHCC had 1.06 more GP visits in the year than those without; the number of visits initially decreased with age before increasing exponentially; for each of their chronic conditions patients made 1.06 more visits in the year (Table 2).

**Table 2:** Model 4— Inclusion of significant patient characteristics

Parameter	Estimate (Visits)	t-Value	*p*-Value
Intercept	2.789	40.63	<0.0001
Female (over male)	0.516	11.56	<0.0001
Age (years)	−0.032	−8.02	<0.0001
Age^2^ (years)	0.00052	9.13	<0.0001
Commonwealth Concession Health Care Card	1.056	14.43	<0.0001
Number of chronic conditions	1.061	38.82	<0.0001

Active patients R-square = 27.59% (*p* < 0.0001) Adjusted R-square = 27.58%

### Model 4A

After adding to the model each of the 28 individual listed chronic conditions (see Table 1), backwards elimination removed all bar seven: hyperlipidaemia; hypertension; peripheral vascular disease; glaucoma; asthma; obesity; and atrial fibrillation. The number of body systems remained significant in this model. Variables removed that had a significance of *p* < 0.5 were: patient rurality (*p* = 0.0328 and effect size of 0.322 extra visits for ‘major city’ patients) and the presence of rheumatoid arthritis (*p* = 0.0360 and effect size of − 0.008).

While statistically significant, the effect of each retained specific individual condition on the GP visit rate was small, ranging from 0.003 fewer visits for a patient with asthma, to 0.014 additional visits for one with atrial fibrillation. This model accounted for 28.40% of the variance in the number of times active patients saw a GP in the previous year, with an adjusted R-square of 28.37% (Table 3).

**Table 3:** Model 4A: Patient characteristics and individual morbidities included

Parameter	Estimate (Visits)	t-Value	*p*-Value
Intercept	2.735	40.14	<0.0001
Female (over male)	0.495	11.04	<0.0001
Age (years)	−0.030	−7.52	<0.0001
Age^2^ (years)	0.0005	8.59	<0.0001
Commonwealth Concession Health Care Card	1.031	14.06	<0.0001
Number of body systems	0.355	4.75	<0.0001
Number of chronic conditions	0.980	14.75	<0.0001
Atrial fibrillation	0.014	4.79	<0.0001
Peripheral vascular disease	0.009	2.65	0.0082
Hyperlipidaemia	−0.012	−9.59	<0.0001
Hypertension	−0.005	−5.56	<0.0001
Glaucoma	−0.008	−3.07	0.0022
Obesity	−0.006	−4.04	<0.0001
Asthma	−0.003	−2.85	0.0044

Active patients R-square = 28.40% (*p* < 0.0001) Adjusted R-square = 28.37%

### Model 4B

Of the 250 GPs who were sent recording forms that included the severity of illness question, 211 (84.4%) completed the sub-study for 6339 patients. Of these, 4610 (72.7%) had at least one chronic condition, for whom a GP-estimated overall severity of illness had been requested. GPs reported severity for 4461 patients (96.8% of those eligible). The average active patient with at least one chronic condition had an overall severity of illness score of 3.5/10.

After retesting the variables from Model 4 on this sub-sample, backwards elimination removed: patient sex; patient advantage/disadvantage; number of body systems; and patient rurality. This model accounted for 19.97% of the variance (R-square) in active patients’ GP visit rate. The adjusted R-square was similar at 19.88%.

After adding severity of illness to the model, the same variables were removed by backwards elimination, as was severity of illness. Severity of illness was the last variable to be removed with a *p*-value of 0.0139 and an effect size of 0.172. The final model is presented in Table 4.

**Table 4:** Model 4B: Severity of illness included

Parameter	Estimate (Visits)	t-Value	*p*-Value
Intercept	3.797	7.95	<0.0001
Age (years)	−0.065	−3.33	0.0010
Age^2^ (years)	0.0009	3.97	<0.0001
Commonwealth Health Care Card	1.744	6.71	<0.0001
Indigenous	−2.310	−2.65	0.0088
Number of chronic conditions	0.817	9.49	<0.0001

Active patients R-square = 19.97% (*p* < 0.0001) Adjusted R-square = 19.88%

## Discussion

Number of chronic conditions was the best predictor of GP visit rate in the previous 12 months, far better than the age and sex of the patient combined. The number of chronic conditions alone accounted for 92.4% of the variance explained by a model including all other significant patient characteristics and 89.7% of a model including all significant patient characteristics and the presence or absence of individual chronic conditions.

The model explaining the most variance included significant patient characteristics, the number of chronic conditions, the number of body systems, and the presence/absence of seven specific chronic conditions. While statistically significant, the effects that the presence of specific chronic conditions had on patient visit rate were so small they are unlikely to be clinically significant. For example, atrial fibrillation had the largest effect size, but a patient would need to have had this condition for 70 years before it resulted in one extra GP visit. We therefore conclude that the most practical parsimonious model is Model 4, which includes patient age and sex, the number of chronic conditions and whether the patient held a CCHCC. This more practical model accounted for 97.1% of the variance explained by the larger model.

The number of body systems was almost as useful in predicting the GP visit rate as the number of chronic conditions (Model 2 c.f. Model 1). This suggests that body systems can be used in lieu of individual chronic conditions when they are not available or there is concern around the robustness of the data.

The number of body systems was removed from our final parsimonious model (Model 4) as it did not significantly explain any more variance than already explained by the count of individual chronic conditions. However, the number of body systems remained significant in the model that included adjustment for the presence/absence of specific conditions (Model 4A). Further investigation is required to assess why body systems were significant in Model 4A, especially since the effect size of the presence of specific individual conditions was so small.

Our results largely reflect those of Knox et al. [10] which found that the number of chronic conditions, patient age and holding a CCHCC all increased the patient GP visit rate. However, unlike Knox et al. we found in our much larger sample that, after adjusting for all other confounding variables, female patients attended more often than males. This is likely a reflection of higher attendance rates of younger women, often for reproductive issues [21], many of which would not usually be classed as chronic conditions.

In Australia, the GP workforce is maldistributed, with fewer GPs in rural and remote than in metropolitan areas [2], The Government currently funds several initiatives to attract GPs to rural/remote areas [22]. We did not find rurality to be a significant predictor of GP visits at our *p* < 0.01 level. However, if we considered it significant at the *p* < 0.05 level, it showed that patients in rural areas attended less often. Lower visit rates may reflect restricted access to care caused by the current GP shortages in rural areas [2]. Including rurality in any model to predict required GP workforce or to calculate capitation payment levels would exacerbate rural patient healthcare disadvantage and would be antithetical to Government initiatives currently in place. For similar reasons no model should pay less for the care provided to Indigenous patients even though Indigenous status was a significant predictor of fewer GP visits in Model 4B. It is known that Indigenous patients face additional barriers to care to those facing non-Indigenous patients [23]. It is likely that if the barriers to GP services were removed for both rural and Indigenous patients they might attend as often as their metropolitan and non-Indigenous peers.

This raises the possibility that changes to the relative number of GPs providing services may affect the number of times patients visit a GP in the future. However, we believe it is appropriate to use the number of services provided with the number of GPs over the study period to inform the Health Care Home remuneration and to act as a baseline for workforce planning.

Severity of illness did not significantly add to our model at the *p* < 0.01 level. However, if included at the *p* < 0.05 level, its effect on GP visit rate was minimal, with a patient needing a 6-point increase in their overall severity of illness to generate one more GP visit in the year. Further, it may be difficult for a GP to objectively judge a patient’s severity of illness knowing they will be reimbursed at a higher rate if the patient is classed as more severe.

The Health Care Homes model will probably result in the transfer of some services currently provided by GPs, to other health professionals in the team. While our model predicts the number of GP visits by a patient over a year, in the Health Care Homes model it is likely to represent overall patient demand for services from general practices.

This study does have a limitation. Our estimate of the average number of GP visits for active patients (4.54) was significantly lower than the average number of Medicare GP consultation items claimed by people who made at least one claim (6.8 in 2014–15) [11]. As discussed in our earlier paper, this means that our GPs and patients were likely to have under-reported the number of GP visits in the previous 12 months [7]. This could be due to the patient seeing another GP that they had forgotten and/or did not wish to disclose to the current GP. If the under-reporting was evenly and proportionally spread among high and low attenders, it would be possible to weight the results of our model up to reflect the observed number of GP visits. However, if the under-reporting was skewed in some way, it is unlikely that reweighting the data would be accurate. The final model should be validated on another independent data source.

## Conclusions

While there are multiple factors that influence the number of times a patient sees a GP in a year, our study found the most parsimonious model included patient age and sex, the number of chronic conditions, and whether the patient holds a Commonwealth Concession Health Care Card. The results of this study will assist with workforce planning and capitation payment trial for GP care of diagnosed chronic conditions in patients enrolled in the Health Care Home trial. Further research is planned to test whether this model also predicts patient complexity of care.

## Abbreviations

ABS: Australian bureau of statistics
ASGS: Australian statistical geography standard
BEACH: Bettering the evaluation and care of health
CCHCC: Commonwealth concession health care card
CIRS: Cumulative illness rating scale
DVA: Department of veteran affairs
GP: General practitioner
ICD-10: International classification of disease version 10
ICPC-2: International classification of primary care version 2
ISRAD: Index of relative socio-economic advantage and disadvantage
